# Trp53 controls chondrogenesis and endochondral ossification by negative regulation of TAZ activity and stability via β-TrCP-mediated ubiquitination

**DOI:** 10.1038/s41420-022-01105-2

**Published:** 2022-07-12

**Authors:** Yang Li, Shu-ting Yang, Shuying Yang

**Affiliations:** 1 Department of Basic & Translational Sciences, School of Dental Medicine, University of Pennsylvania, Philadelphia, PA 19104 USA; 2 The Penn Center for Musculoskeletal Disorders, School of Medicine, University of Pennsylvania, Philadelphia, PA 19104 USA; 3 Center for Innovation & Precision Dentistry, School of Dental Medicine, School of Engineering and Applied Sciences, University of Pennsylvania, Philadelphia, PA 19104 USA

**Keywords:** Bone development, Differentiation

## Abstract

Transformation-related protein 53 (Trp53) is a critical regulator of cell fate determination by controlling cell proliferation and differentiation. Ablation of Trp53 signaling in osteoblast lineages significantly promotes osteogenesis, bone formation, and bone remodeling. However, how Trp53 regulates chondrogenesis and endochondral bone formation is undefined. In this study, we found that Trp53 expression gradually decreased in tibia growth plates during embryonic development in vivo and during chondrogenesis in vitro. By deleting Trp53 in chondrocyte lineage using Col2-Cre transgenic line, we found that loss of Trp53 in chondrocytes significantly increased growth plate growth and bone formation by increasing chondrocyte proliferation, matrix production and maturation, and bone dynamic formation rate. Mechanistically, our data revealed loss of Trp53 significantly promoted TAZ transcriptional activity through inhibition of TAZ phosphorylation and nuclear translocation, whereas its activity was pronouncedly inhibited after forced expression of Trp53. Furthermore, Co-IP data demonstrated that Trp53 associated with TAZ. Moreover, Trp53 decreased the stability of TAZ protein and promoted its degradation through β-TrCP-mediated ubiquitination. Ablation of TAZ in Col2-Cre;Trp53^f/f^ mice rescued the phenotypes of enhanced chondrogenesis and bone formation caused by Trp53 deletion. Collectively, this study revealed that Trp53 modulates chondrogenesis and endochondral ossification through negative regulation of TAZ activity and stability, suggesting that targeting Trp53 signaling may be a potential strategy for fracture healing, heterotopic ossification, arthritis, and other bone diseases.

## Introduction

Chondrogenesis is a critical process of skeletal development, which drives the long bone formation through modulating the formation and extension of the cartilage growth plate, and terminates with endochondral ossification [1–3]. Previous studies showed that over 80% of the skeleton volume is formed by induction of endochondral bone formation [1, 2]. During endochondral ossification, chondrocytes undergo multiple steps including proliferation, differentiation, and mineralization; and the mineralized extracellular matrix could be invaded by osteoclasts and osteoblasts [4, 5] and eventually deposited on the cartilage matrix to form bone [4, 6]. Dysfunction of endochondral ossification causes abnormal skeletal development and some skeletal disease such as chondrodysplasias and osteoarthritis [7, 8]. In recent decades, bone diseases become a more severe public health issue with an increasing prevalence and a high impact on morbidity worldwide [9]. However, clinical management of these diseases is constrained by the severity of the diseases due to the lack of precise molecular dissections. Therefore, elucidating the molecular mechanisms involved in chondrogenesis and endochondral ossification is important for the improving therapeutic strategies for cartilage and bone-related diseases.

Recently, mounting evidence indicated that the transcription factor Trp53 as a guardian directs the function of mesenchymal stem cells (MSCs) and cell fate determination by controlling cell cycle, differentiation, and its target genes’ expression [8, 10, 11]. In bone, ablation of Trp53 signaling in MSCs and osteoblasts is closely associated with the significant enhancement of osteogenesis and skeletal development [10, 12, 13]. Moreover, Trp53 was also found to be involved in bone remodeling through negative regulation of osteoblast-dependent osteoclastogenesis [10, 12]. In addition, Trp53 expresses in chondrocytes and regulates the onset and progression of some cartilage-related diseases such as chondrosarcoma, osteoarthritis, and rheumatoid arthritis [14–17], indicating that Trp53 may play a pronounced role in chondrogenesis. However, the role of Trp53 in chondrocytes and the precise molecular mechanisms of Trp53 controlling the chondrogenesis and endochondral ossification are largely undefined.

Hippo pathway is considered as a crucial regulator of the lineage commitment of MSCs and skeletal development through the regulation of cell proliferation and apoptosis [1, 18–20]. A few studies have reported that Trp53 and the Hippo pathway are “functionally” and “physically” linked, highlighting the dire consequence of impaired coordination [21–23]. Transcriptional co-activator with PDZ-binding domain (TAZ) is an important downstream effector of Hippo pathway and involved in the regulation of the pathway [1, 18, 19]. TAZ expression level is critical in determining the cell fate [24]. Our previous findings revealed that TAZ is a positive regulator of the chondrogenesis and endochondral ossification [1]. Accordingly, Trp53 was also reported to be required for stem cell self-renewal and cell fate determination [23, 25–27]. Of note, mutant Trp53 enhances TAZ stability by regulating WASP-interacting protein (WIP) signaling in breast cancer and glioblastoma [28]. However, it remains unknown whether Trp53 governs chondrogenesis and endochondral ossification through regulating TAZ signaling.

In this study, we explored the role and molecular mechanism by which Trp53 regulates chondrogenesis and endochondral ossification. Our data revealed that Trp53 is an endogenous inhibitor in chondrogenesis and endochondral ossification by negatively regulating the activity and stability of TAZ via β-TrCP-mediated ubiquitination in chondrocytes.

## Results

### Trp53 signature is gradually decreased during chondrogenesis and embryonic bone development

**Fig. 1:**
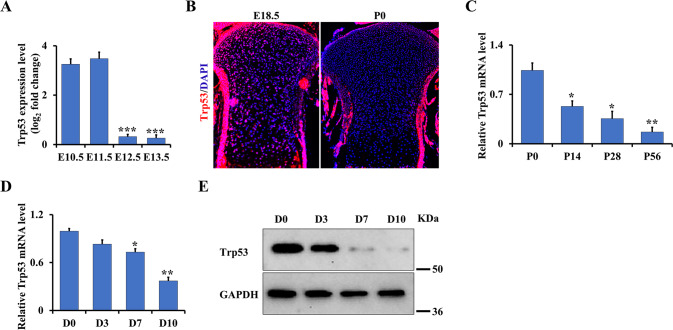
Trp53 signature is gradually decreased during chondrogenesis and embryonic bone development. **A** Trp53 expression was identified in the primary chondrocytes using the publicly available early murine limb development data from GSE30138 [29] as indicated. E: embryonic day. **B** Representative immunofluorescent image of Trp53 in the tibia from wild-type mice as shown. P0: the newborn. Scale bars, 75 μm. **C** Trp53 expression in cartilage as indicated. P: postnatal day. **D**, **E** Trp53 expression was determined by qRT-PCR and western blot after culture as indicated time in chondrogenic differentiation medium. D: day. *P < 0.05, **P < 0.01, ***P < 0.001.

To investigate the function of Trp53 in chondrogenesis, we first identified the expression pattern of Trp53 during early murine limb development by analyzing the publicly available database (GSE30138) [29]. Interestingly, we found the Trp53 expression was highly expressed at E10.5 and E11.5, which was significantly decreased at E12.5 and E13.5 (Fig. 1A). Consistently, the endogenous expression pattern of Trp53 also revealed a decrease in the tibia growth plate of wild-type mice at newborn (P0) compared to that at embryonic day (E) 18.5 (Fig. 1B), indicating that Trp53 expression is decreased during embryonic bone development. Moreover, qPCR analysis also displayed a gradual decrease in Trp53 expression during postnatal bone development from P14, P28 to P56 (Fig. 1C). To further determine the function of Trp53 during chondrogenesis, we next isolated primary chondrocytes from the limb buds of wild-type mice embryos at E15.5 and performed the micromass culture to identify its expression during chondrogenesis. Similar to its expression pattern in embryos, qPCR and western blot data also showed a significant reduction of Trp53 expression during chondrogenic differentiation (Fig. 1D, E). Taken together, these data indicated that Trp53 may play a critical role in bone development.

### Ablation of Trp53 in chondrocytes increases bone formation and promotes extension of the growth plate

**Fig. 2:**
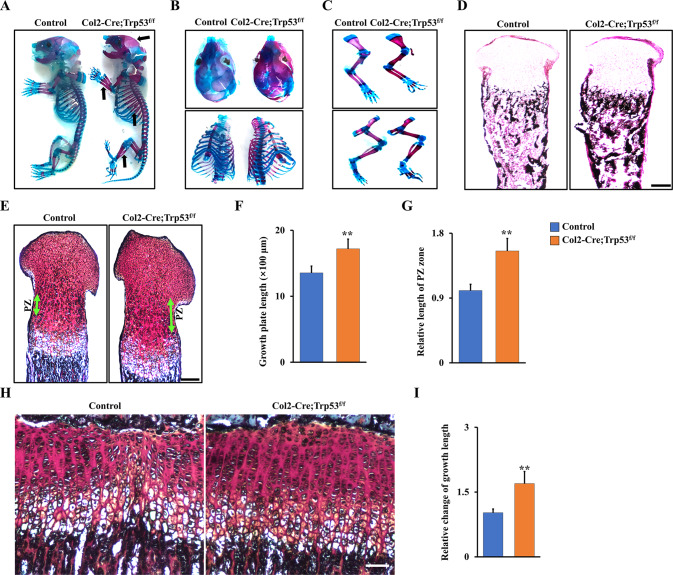
Trp53 is required for normal skeletal development. **A**–**C** Representative whole-mount skeletal-stained images as indicated at P0. The black arrows direct to the regions of enhanced bone mass at P0. **D** Representative von Kossa-stained images in the tibiae as indicated at P0. *N* = 3. Scale bars, 75 μm. **E** Representative Safranin O-stained images as indicated at P0. Green arrow directs to the proliferation zone (PZ). Scale bars, 100 μm. **F** Quantitative analysis of growth plate length in the newborn tibiae as indicated. *N* = 3. **G** Relative analysis of PZ’s lengths in the newborn tibiae as shown. *N* = 3. **H**, **I** Representative Safranin O-stained images as indicated at 1 month (**H**). Scale bars, 5 μm. The fold change of the growth plate length from (**H**) was identified (**I**). *N* = 3. **P < 0.01.

To investigate the potential contributions of Trp53 to chondrogenesis in vivo, we generated the Col2-Cre;Trp53^f/f^ mice. qRT-PCR analysis confirmed that Trp53 was efficiently deleted in chondrocytes instead of osteoblasts from Col2-Cre;Trp53^f/f^ mice compared to the controls (Fig. S1A, B, Supporting Information). Western blot data also showed the protein level of Trp53 had no pronounced change in the osteoblasts due to Trp53 ablation in chondrocytes (Fig. S1C, Supporting Information). Next, we characterized the skeletal phenotype of Col2-Cre;Trp53^f/f^ mice. Interestingly, we found that the Col2-Cre;Trp53^f/f^ newborn pups exhibited a significant increase in a mineralized bone matrix of the calvarium, sternum, ribs, and limbs (Fig. 2A–C). Moreover, Von Kossa staining results also showed a remarkable increase in the bone mineralization of the tibia from newborn Col2-Cre;Trp53^f/f^ mice compared to those in the Cre control mice (Fig. 2D). To further study the cause of the enhanced skeletal components, Safranin O/Fast green staining was performed in tibiae at newborn mice. Our data demonstrated that the lengths of the growth plate and proliferation zone (PZ) were significantly increased in the Col2-Cre;Trp53^f/f^ mice compared to the age-matched controls (Fig. 2E, F). Intriguingly, quantitative results showed that the relative length of PZ zone increased by 1.55-fold in Col2-Cre;Trp53^f/f^ group compared to the Cre control group (Fig. 2G), suggesting that deletion of Trp53 significantly promoted chondrocyte proliferation, as evidenced by increased growth plate at 1-month-old Col2-Cre;Trp53^f/f^ mice (Fig. 2H, I).

### Trp53 deficiency in chondrocytes causes osteopetrosis phenotype with increased bone formation and mineralization

**Fig. 3:**
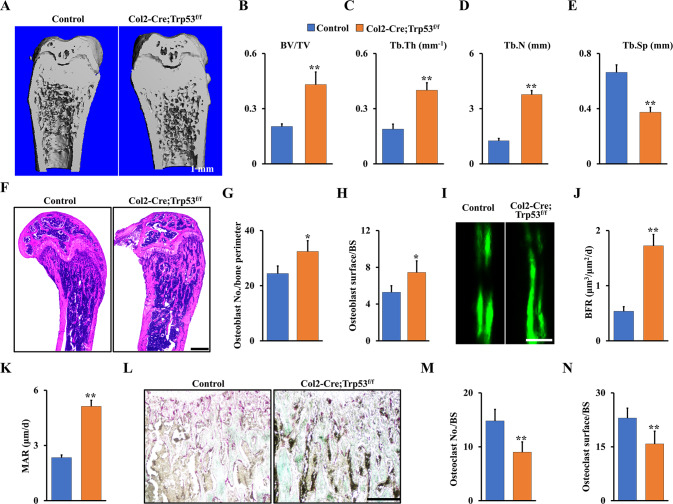
Trp53 deficiency in chondrocytes promotes bone formation. **A** Representative micro-CT reconstruction as indicated at 2 months. *N* = 5. Scale bars, 1 mm. **B**–**E** Histomorphometric analysis of bone parameters from (**A**) as shown. **F** Representative H&E images of femur sections from 2-month-old Col2-Cre;Trp53^f/f^ mice and controls. *N* = 5. Scale bar, 1 mm. **G** Osteoblast number per bone perimeter. **H** Osteoblast surface per bone surface (osteoblast surface/BS). **I**–**K** Representative image of double calcein labeling in the tibia from 2-month-old Col2-Cre;Trp53^f/f^ mice and controls. Scale bar, 50 μm. **L** TRAP staining. Scale bar, 50 μm. **M** Osteoclast No./BS. **N** Osteoclast surface/BS. **P < 0.05.

To further confirm the function of Trp53 in skeletal development, we examined the bone mass of 2-month-old Col2-Cre;Trp53^f/f^ mice and age-matched controls by X-ray and micro-CT. As expected, we found loss of Trp53 in chondrocytes significantly increased the bone mass (Fig. 3A; Fig. S2, Supporting Information). The values of bone volume per total volume (BV/TV), trabecular thickness (Tb.Th), and trabecular number (Tb.N) were respectively increased 2.21-, 1.96-, and 3-fold, and the value of trabecular separation (Tb.Sp) was decreased ~41% in femurs from the Col2-Cre;Trp53^f/f^ mice compared to those in the controls (Fig. 3B–E). This finding was further confirmed by H&E staining (Fig. 3F) and analysis of osteoblast number and osteoblast surface (Fig. 3G, H) in 2-month-old the Col2-Cre;Trp53^f/f^ mice. To further examine bone dynamic changes, we conducted histomorphometric analysis of the femur metaphysis and found that the Col2-Cre;Trp53^f/f^ mice displayed a significantly increased BFR and MAR compared to those in the controls (Fig. 3I–K). To identify whether loss of Trp53 in chondrocytes affects osteoclastogenesis, we next performed TRAP staining in Col2-Cre;Trp53^f/f^ mice and controls. Our data showed a significant decrease in osteoclastogenesis in Col2-Cre;Trp53^f/f^ mice compared to the controls (Fig. 3L–N), indicating that the enhanced bone mass may be partly caused by impaired osteoclastogenesis. Overall, these data demonstrated that Trp53 is required for chondrogenesis and endochondral ossification.

### Loss of Trp53 in chondrocytes promotes chondrocyte proliferation, matrix production, and maturation

**Fig. 4:**
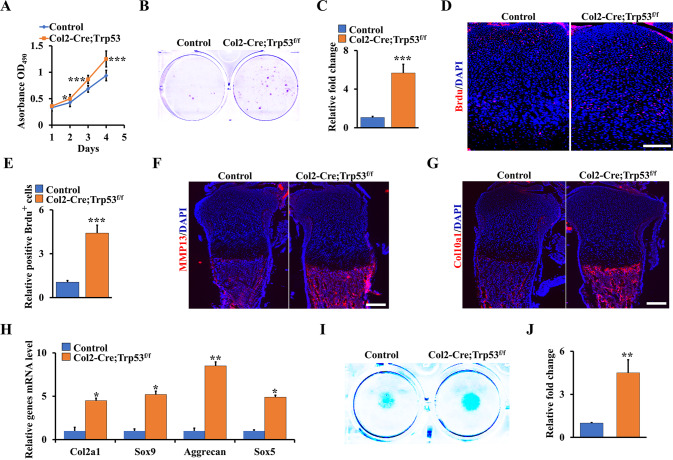
Trp53 is indispensable for chondrocyte proliferation, matrix production, and maturation. **A** The proliferation of primary chondrocytes from Col2-Cre;Trp53^f/f^ mice and controls was identified at different times as shown by WST-1 assay. **B**, **C** Colony formation (**B**) and quantitative analysis of the colony numbers as shown (**C**). **D**, **E** BrdU staining (D) and quantitative analysis of BrdU^+^ cells (**E**) in the tibiae from Col2-Cre;Trp53^f/f^ mice and control at P0. Scale bars, 25 μm. **F** MMP13 staining at P0 tibiae as indicated. Scale bars, 75 μm. **G** Representative fluorescence images of Col10a1 at P0 tibiae as indicated. Scale bars, 75 μm. **H** The mRNA level of chondrocyte differentiation markers in primary chondrocytes as indicated. **I**, **J** The primary chondrocytes were cultured by micromass in chondrogenic medium as indicated. After 7 days’ culture, the macromass were stained by the alcian blue solution as shown. The relative fold change of intensity was identified (**J**). *P < 0.05, **P < 0.01, ***P < 0.001.

Given that loss of Trp53 in chondrocytes enhanced the chondrogenesis and endochondral ossification, we next tested whether Trp53 deficiency could evoke the formation and maturation of chondrocytes in vitro. We first isolated the primary chondrocytes from Col2-Cre;Trp53^f/f^ mice and controls and explored the effect of Trp53 on proliferation in these cells. The result of WST-1 assay showed that deletion of Trp53 greatly increased the proliferation rate of chondrocytes (Fig. 4A). Concomitantly, the colony numbers of Trp53-mutant cells also remarkably increased compared to those in the control cells (Fig. 4B, C). By analysis of the proliferating cells in the growth plate of tibia of Col2-Cre;Trp53^f/f^ newborns, we found loss of Trp53 in chondrocytes significantly increased cell proliferation (Fig. 4D, E). Moreover, the expression levels of collagen type X (Col10a1) and matrix metalloproteinase 13 (MMP13) were dramatically increased in chondrocytes from the Col2-Cre;Trp53^f/f^ newborns compared to those from Cre control newborns (Fig. 4F, G). Consistently, qRT-PCR results further confirmed that loss of Trp53 in chondrocytes promoted chondrocyte differentiation and maturation (Fig. 4H), as evidenced by the micromass culture staining in Trp53-mutant primary chondrocytes and controls (Fig. 4I, J).

### Trp53 associates with TAZ, decreases the stability of TAZ protein and promotes its degradation through β-TrCP-mediated ubiquitination in chondrocytes

**Fig. 5:**
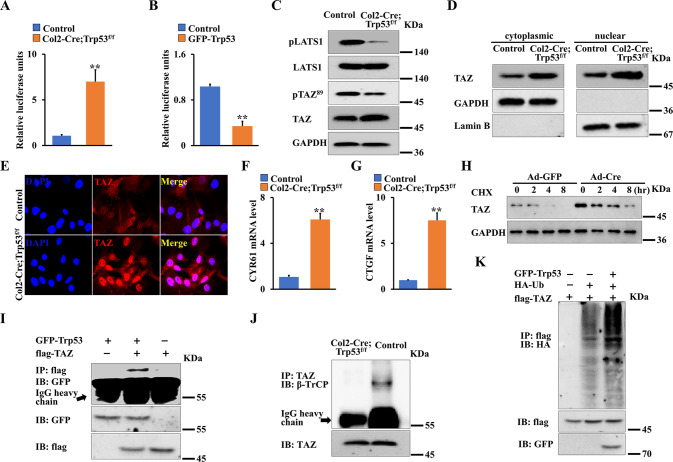
Trp53 regulates the stability and activity of TAZ in chondrocytes. **A** The luciferase activity was measured by Promega® Luciferase Assays kit in primary chondrocytes as indicated. **B** Luciferase activity was measured in the primary chondrocytes the following co-transfection with pEGFP (control), pEGFP-Trp53, respectively. **C** Whole protein lysates of primary chondrocytes as indicated were detected by western blot. **D** TAZ expression in cytoplasm and nucleus as indicated. **E** Representative fluorescence images of TAZ in primary chondrocytes as indicated. Scale bars, 25 μm. **F**, **G** mRNA level of CYR61 and CTGF in primary chondrocytes as indicated. **H** Primary chondrocytes from Trp53^f/f^ mice were transfected with Ad-Cre or Ad-GFP, after 24 h, the cells were treated for different time as indicated with 50 μg/mL cycloheximide (CHX). The TAZ expression was identified by western blot. **I** Co-IP experiments were performed after transfection of plasmid GFP-Trp53 or/and flag-TAZ in HEK293T cells as indicated. **J** Co-IP experiments in primary chondrocytes as indicated. **K** Western blot analysis of TAZ ubiquitination demonstrating that Trp53 is necessary for TAZ ubiquitination. **P < 0.01.

Accumulating evidence showed that TAZ is a critical regulator of skeletal development and bone mass [1, 18, 19]. In addition, our previous findings showed loss of TAZ in chondrocytes inhibits chondrogenesis and skeletal development [1]. To determine whether Trp53 affects chondrogenesis through regulating TAZ signaling, we first identified the transcriptional activity of TAZ when Trp53 was deleted or overexpression in primary chondrocytes. As shown in Fig. 5A, B, loss of Trp53 promoted, whereas forced expression of Trp53 markedly inhibited the transcriptional activity of TAZ. To further confirm the interplay between Trp53 and TAZ activity, we next evaluated the phosphorylation of TAZ (pTAZ) and large tumor suppressor kinase 1 (Lats1) (pLats1), which acts upstream of TAZ to enhance TAZ phosphorylation. As expected, we found that loss of Trp53 inhibited the protein levels of pLats1 and pTAZ (Fig. 5C). Moreover, we found that TAZ nuclear translocation and the expression of TAZ target genes including Cyr61 and CTGF were significantly increased in Trp53-deficient primary chondrocytes compared to those in the control cells (Fig. 5D–G). In addition, deletion of Trp53 in chondrocytes increased the expression of TAZ compared to that in the control after the cells were treated with the protein synthesis inhibitor cycloheximide, indicating that Trp53 could promote TAZ stability (Fig. 5H). It has been reported that E3 ligase β-TrCP controls the stability and degradation of TAZ protein [30, 31]. We next examined whether loss of Trp53 enhanced TAZ stability through β-TrCP-mediated ubiquitination of TAZ. Interestingly, we found Trp53 did associate with TAZ (Fig. 5I). Moreover, our Co-IP results showed that TAZ couldn’t bind with β-TrCP in Trp53-mutant primary chondrocytes, on the contrary, TAZ showed a binding to β-TrCP protein in the normal primary chondrocytes (Fig. 5J), indicating that loss of Trp53 may inhibit β-TrCP-mediated ubiquitination of TAZ. To further test whether Trp53 affects TAZ stabilization through the β-TrCP, we co-transfected flag-TAZ with/without GFP-Trp53 and HA-Ub in HEK293T. Western blot results revealed that TAZ ubiquitination was markedly enhanced after overexpression of Trp53 (Fig. 5K). Taken together, these findings suggested that loss of Trp53 promotes the stability and nuclear translocation of TAZ in chondrocytes.

### Deletion of TAZ in Col2-Cre;Trp53^f/f^ mice attenuates the enhanced bone formation caused by loss of Trp53 in chondrocytes

**Fig. 6:**
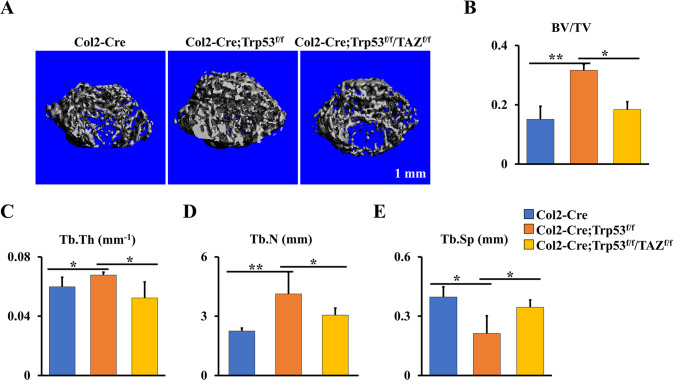
Trp53 modulates chondrogenesis and endochondral ossification through TAZ signaling. **A** Representative micro-CT reconstructions of tubular bone of 1-month-old Col2-Cre;Trp53^f/f^, Col2-Cre;Trp53^f/f^/TAZ^f/f^ mice and age-matched controls. Scale bars, 1 mm. **B**–**E** The histomorphometric analysis of bone parameters were performed as indicated. *P < 0.05, **P < 0.01.

**Fig. 7:**
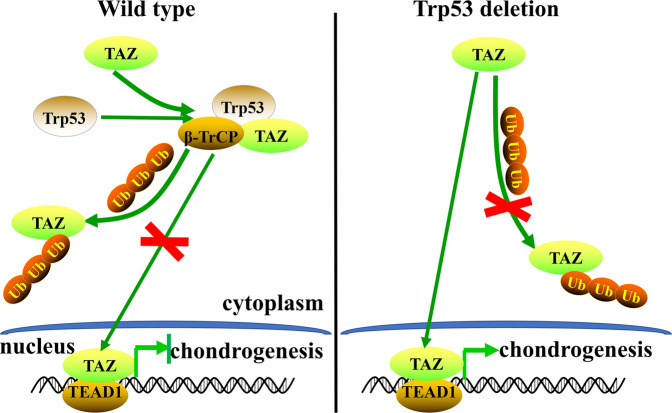
Trp53 modulates chondrogenesis and endochondral ossification through TAZ signaling. In normal conditions, Trp53 interacts with TAZ to inhibit its nuclear translocation, and promotes its degradation through β-TrCP-mediated ubiquitination, which ultimately causes a decreased chondrogenesis.

To further assess the regulation of TAZ signaling in Trp53-mediated chondrogenesis and endochondral ossification, we bred TAZ^flf^ mice with Col2-Cre;Trp53^f/f^ mice to generate a double knockout mouse model Col2-Cre;Trp53^f/f^/TAZ^f/f^. As expected, we found that ablation of TAZ signaling in the Col2-Cre;Trp53^f/f^ mice partly attenuated the increased bone formation caused by Trp53 deficiency in chondrocytes (Fig. 6A). The femurs from the 1-month-old Col2-Cre;Trp53^f/f^/TAZ^f/f^ mice lost ~42% of BV/TV, 23% of Tb.Th, and 26% of Tb.N, and Tb.Sp showed a 1.63-fold increase compared to those in the age-matched Col2-Cre;Trp53^f/f^ mice (Fig. 6B–E). Taken together, our data suggested that Trp53 modulates chondrogenesis and endochondral ossification through TAZ signaling (Fig. 7).

## Discussion

Previous findings showed that Trp53 plays crucial role in osteoblast-dependent bone formation and remodeling [10, 12, 13]. However, the role and mechanism of Trp53 in the regulation of chondrogenesis and endochondral ossification are largely undefined. In this study, we found Trp53 controls chondrogenesis and endochondral ossification by negative regulation of TAZ activity and stability.

Recent studies showed that Trp53 functions as a transcriptional factor to be involved in cell proliferation and differentiation such as osteogenesis, myogenesis, adipogenesis, and neurogenesis through the regulation of dozens of target genes’ expressions and functions [32–34]. For instance, abnormal activity and expression of Trp53 are observed to be closely related to the developmental defects and premature aging in humans [27]. Deletion of Trp53 in MSCs and osteoblasts has been reported to accelerate osteogenic differentiation and cause a significantly increased bone formation by upregulation of the core osteogenic transcription factors including Runx2 and Osterix [10, 13, 35]. In addition, high expression of Trp53 occurs along with the progression of osteoarthritis and rheumatoid synovium compared to the normal chondrocytes [14, 36]. Our results showed deletion of Trp53 in chondrocyte lineage significantly increased bone mass accompanied by enhanced chondrocyte proliferation and maturation. Moreover, loss of Trp53 in chondrocytes contributed to extending of the growth plate along with increased expression of chondrocyte marker Col10a1 and MMP13. In consistent with our results, global deletion of Trp53 elevated cell proliferation, osteogenesis and bone formation [10, 13]. Mizuhashi et al. reported that parathyroid hormone-related protein (PTHrP)-positive resting chondrocytes could undergo hypertrophy and become marrow stromal cells and osteoblasts [37], however, our results did not show a significant difference in osteoblastic Trp53 expression level compared to the cells without Trp53 deletion. There are two possible reasons. One is that the PTHrP+ osteoblasts directly derived from chondrocytes are not dominant in the cortical bone. Osteoblasts may be also derived from PTHrP- or non-chondrocytes. One the other hand, Col2+ cell lineage tracing studies from our and other groups have shown that Col2+ cells dramatically decreased starting from one month [38, 39]. This is also a possible reason to explain why osteoblasts have no Trp53 deletion. The clear answer needs to be further determined.

Interestingly, numerous studies have reported that loss of Trp53 in osteoblast progenitor cells causes increased bone formation, spontaneous osteosarcoma and lung metastasis, and eventually dies around 10 months [40]. However, we didn’t find tumor formation after the loss of Trp53 in chondrocytes. It may be due to two reasons. One is that Trp53 expression pattern in chondrocytes is different from that in osteoblasts. Wang et al. reported that Trp53 expression significantly increased starting from day 4 during osteoblast differentiation [10]. However, our results showed that Trp53 expression is gradually decreased during chondrogenesis. Another reason is that Col2-positive cells are decreased with age [41]. While accumulating evidence suggested that aging was a major risk factor of tumorigenesis that displayed an inevitable time-dependent manner [42, 43]. Thus, the effect of Trp53 alone in chondrocytes on tumor formation is limited due to its non-matched expression timeline with tumorigenesis.

Hippo pathway is a highly conserved regulator of development and organ size control [1, 20]. Recent evidence demonstrated that Trp53 and the Hippo pathway could cooperate with each other to regulate the cell fate [21–23]. It is well-known that TAZ as a downstream effector of Hippo pathway plays a critical function of stemness and bone development [18–20], and our previous study showed that loss of TAZ in chondrocytes causes growth retardation and impairs chondrogenesis and endochondral ossification [1]. Here, we found that loss of Trp53 displayed an increased transcriptional activity of TAZ, whereas forced expression of Trp53 inhibited its activity. Moreover, our data also showed that loss of Trp53 inhibited TAZ phosphorylation at Ser 89 through inhibiting its upstream kinase Lats1 phosphorylation to enhance its activity. These findings were supported by a wealth of accumulated studies demonstrating that loss or mutant of Trp53 increases TAZ nuclear translocation and elevates its transcriptional activity [23, 28]. Liu et al. reported the stability and degradation of TAZ are mainly controlled by E3 ligase β-TrCP [31]. Our data showed that interaction of Trp53 with TAZ decreased the stability of TAZ protein and promoted its degradation through β-TrCP. In vivo, after ablation of TAZ signaling in Col2-Cre;Trp53^f/f^ mice, we found the enhanced chondrogenesis and endochondral ossification caused by loss of Trp53 in chondrocytes were partly prohibited.

Collectively, this study provides a new understanding of Trp53 regulation in endochondral ossification and identifies Trp53 and TAZ may be a potential target for regulation of skeletal development and diseases.

## Materials and methods

### Animals

The Trp53^f/f^ and Col2-Cre mice were purchased from the Jackson Laboratory (USA). The floxed TAZ mice were generated by our lab. All the mice were bred and maintained under standardized conditions in animal facilities at the University of Pennsylvania.

### Antibodies and reagents

Antibodies against Trp53 (#2524; dilution 1:1000), LAST1 (#3477; dilution 1:1000), pLAST1 (#8654; dilution 1:1000), TAZ (#83669; dilution 1:1000), pTAZ (#59971; dilution 1:1000), HA (#5017; dilution 1:1000), GAPDH (#5174; dilution 1:1000); cycloheximide (CHX) and insulin-transferrin-sodium selenite media supplement (ITS Supplement) were purchased from Cell Signaling Technology. BrdU (#sc-32323; dilution 1:1000), β-TrCP (#sc-390629; dilution 1:1000), GFP (#sc-9996; dilution 1:1000) and flag (#sc-7945; dilution 1:1000) antibodies were obtained from Santa Cruz Biotechnology. Col10a1 (#14-9771-80; dilution 1:1000) antibody, EDTA-free cocktail inhibitor tablets, calcein and BrdU labeling were obtained from Fisher Scientific™. The Transfection Reagent FuGENE^®^ HD was ordered from Promega (USA). The plasmids GFP-Trp53, flag-TAZ, and HA-Ub were from Addgene (USA).

### Cells and cell culture

The isolation of primary chondrocytes was performed as we previously described [1]. Briefly, the E15.5 limb buds from wild-type, Col2-Cre or Col2-Cre;Trp53^f/f^ mice were first dissociated in trypsin solution (Fisher Scientific™, USA) for 30 min at 37 °C. Subsequently, the cells were washed with sterile phosphate buffered saline (PBS) and collected (primary chondrocytes). The primary chondrocytes and HEK293T were cultured in α-MEM and DMEM medium with 10% FBS, respectively. Micromass cultures were performed as we previously described [1].

### qRT-PCR

Two micrograms of the total RNA extracted by TRIzol reagent (Invitrogen, USA) was retro-transcribed into cDNA using the PrimeScript™ RT Reagent Kit (Invitrogen, USA). The cDNA as the templates was subsequently performed the qRT-PCR with SYBR reagents. The primers used in this study were listed in Supplementary information, Table S1.

### WST-1 and colony formation assay

For the proliferative rate of primary chondrocytes, briefly, logarithmically growing cells were trypsinized, and 5 × 10^3^ primary chondrocytes were seeded in 96-well plates. After indicated time in culture, the OD_490_ was measured using WST-1 kit [1, 44]. For the colony formation, briefly, 5 × 10^3^ primary chondrocytes were seeded and cultured in 6-well plates. After 5 days of culture, based on the crystal violet staining, the colony numbers were counted and analyzed.

### Whole-mount skeletal staining

Briefly, the newborn pups were euthanized and fixed by 100% ETOH for overnight at room temperature. After the fixation, the newborn pups were treated by acetone for 12 h, and then stained by the alcian blue solution and alizarin red solution respectively as we previously reported [1, 45].

### Micro-CT and X-ray analyses

The femurs from 1- or 2-month-old Col2-Cre, Col2-Cre;Trp53^f/f^, Col2-Cre;Trp53^f/f^/TAZ^f/f^ and age-matched controls were analyzed by micro-CT system at facility core of University of Pennsylvania as we previously reported [1, 44, 45]. The X-ray was performed by the Siemens X-ray equipment as we previously reported [1, 44].

### Histology

The femurs and tibiae of Col2-Cre;Trp53^f/f^ and controls were collected and fixed in 4% paraformaldehyde (PFA) for overnight at 4 °C; and then they were decalcified in PBS with 14% ethylene-diamine-tetraacetic acid (EDTA) (pH, 7.4) for 6 weeks. Subsequently, the samples were embedded by paraffin and cut to the 6-μm sections. Hematoxylin & eosin (H&E), Alcian blue, Safranin O/fast green and tartrate-resistant acid phosphatase (TRAP) staining were carried out as we previously described [1, 44–46].

### Calcein labeling

20 mg/kg calcein was carried out at Day 2 and Day 7 before 2-month-old Col2-Cre;Trp53^f/f^ mice and age-matched controls were sacrificed. The tibiae and femurs were collected, fixed in 4% PFA for 12 h at dark, embedded by paraffin, and cut to 6-μm thickness. And then, the bone formation rate per bone surface (BFR, μm^3^/um^−2^ per day) and mineral apposition rate (MAR, μm per day) were observed and analyzed by the OsteoMeasure analysis system as we previously described [1, 45].

### Western blot and Co-IP

Briefly, the primary chondrocytes from Col2-Cre;Trp53^f/f^ mice and controls were lysed with RIPA lysis buffer containing the protein inhibitor cocktail for 15 min on the ice. And then, 25 μg proteins were subjected to SDS-PAGE gel, transferred to the polyvinylidene difluoride (PVDF) membrane (Millipore, USA), and immunoblotted with the corresponding primary antibodies for overnight at 4 °C. After washing for three times with 0.1% TBST (Tween-20 in TBS), the PVDF membranes were incubated with secondary antibody. After incubation for 1 h at room temperature, the membranes were washed by TBST for three times and analyzed by the highly sensitive ECL solution as we previously reported [1, 44, 47]. Co-IP was carried out as we previously reported [1, 44].

### Immunofluorescence

Briefly, the tibial sections at newborn were treated by serial incubations of xylene and ethanol. And then, the sections were blocked with 1% bovine serum albumin (BSA) for 1 h at room temperature and incubated with the corresponding primary antibodies against Trp53 (1:200 dilution) BrdU (1:200 dilution), MMP13 (1:200 dilution) and Col10a1 (1:200 dilution) for overnight at 4 °C. After three times washing with PBS containing 0.1% Triton X-100, the tibial sections were incubated with the secondary fluorescent antibody, stained by 4′,6-diamidino-2-phenylindole (DAPI) and observed as we previously reported [1, 44, 48].

### Bioinformatic analysis

The public RNA-Seq data from GSE30318 [29] were used to determine Trp53 expression at four stages of limb development (from E10.5 to 13.5). All data were downloaded and analyzed by R packages DESeq2 and ClusterProfiler.

### Statistical analysis

The data of this study were analyzed using Student’s t-test and reported as mean ± SEM. two-way ANOVA was used for the statistical evaluations of multiple groups. *P* values < 0.05 were considered significantly.

## Supplementary information

SUPPLEMENTAL MATERIAL

Original Data File

Figure S1

Figure S2

## Data Availability

All data are available upon request.
