# Supplementary material for: Trp53 controls chondrogenesis and endochondral ossification by negative regulation of TAZ activity and stability via β-TrCP-mediated ubiquitination
Source: Cell Death Discov. 2022 Jul 12;8:317. doi: 10.1038/s41420-022-01105-2 (PMC9279315; doi:10.1038/s41420-022-01105-2)
Supplement: Supplementary file 1 — SUPPLEMENTAL MATERIAL [file 41420_2022_1105_MOESM1_ESM.docx]

**Trp53 controls chondrogenesis and endochondral ossification by negative regulation of TAZ activity and stability via β-TrCP-mediated ubiquitination**

Yang Li^1^, Shuying Yang^1^, and Shuying Yang^1,2,3*^

^1^Department of Basic & Translational Sciences, School of Dental Medicine, University of Pennsylvania, Philadelphia, PA 19104, USA

^2^The Penn Center for Musculoskeletal Disorders, School of Medicine, University of Pennsylvania, Philadelphia, PA 19104, USA

^3^Center for Innovation & Precision Dentistry, School of Dental Medicine, School of Engineering and Applied Sciences, University of Pennsylvania, PA 19104, USA

^*^Correspondence: Shuying Yang (shuyingy@upenn.edu)

**Supplementary information, Figure legends**

**Fig. S1. TAZ expression in** **primary chondrocytes and osteoblasts.** (A) Trp53 expression in the primary chondrocytes from Col2-Cre;Trp53^f/f^ mice and control. (B) Trp53 expression in the primary calvarial osteoblasts from Col2-Cre;Trp53^f/f^ mice and control as indicated. (C) Trp53 expression in cortical bone cells. Error bars were the means ± SEM from three independent experiments. ***P* < 0.01.

**Fig. S2. Trp53 deficiency in chondrocytes promotes bone formation.** Representative X-ray images of femurs from 2-month-old Col2-Cre;Trp53^f/f^ mice and control.

**Supplementary information, Table S1**

| **Gene** | **sequence （5'-3'）** | **Gene** | **sequence （5'-3'）** |
| --- | --- | --- | --- |
| GAPDH-F | CCTGGTCACCAGGGCTGCCATTT | Runx2 | GCCGCAGTGCCCCGATTGAG |
| GAPDH-R | CGTTGAATTTGCCGTGAGTGGAG | Runx2 | GTCGGTGCGGACCAGTTCGG |
| TAZ-F | GCAGACATCTGCTTCACCAA | Pthlh-F | TGGAGTGTCCTGGTATTCCTGC |
| TAZ-R | TTCCCTTCTGGGAAGATGTG | Pthlh-R | ACACAGCGCGTTTGAGCCTG |
| TEAD1-F | CCAGATACATCAAACTCAGGACGG | Runx3-F | CTCCTTCCCCAACTATACACCAACC |
| TEAD1-R | GGCGGCTTGAATTTCTCGAACT | Runx3-R | TGTTCTCGCCCATCTTGCCG |
| CTGF-F | GTCTGCGCCAAGCAGCTGGGAGAAC | Col10a1-F | GGGATTCCAGTAAGAGGAGAACAAGG |
| CTGF-R | GCAGTGCACACTCCGATCTTGCG | Col10a1-R | TCCATAGCCTGGCTTGCCTG |
| Cyr61-F | GCCCTCTGGAGGCACCCAAGTGC | Aggrecan-F | CTAGGGTCTTCCCTCACCATCCCC |
| Cyr61-R | GGTCGCAGGGCTGAGTTTTGCTG | Aggrecan-R | CACTTGATTCTTGGGGTGAGGG |
| Sox5-F | GCAGACAGAAAGTGGAAGAGGAGG | Col9a1-F | AGTGAACTGTGTCCCAAGATCAGG |
| Sox5-R | GGCTGAAATTCCTCAGAGTGAGG | Col9a1-R | GTCCTTCGAGATGCAGCCTTC |
| Ihh-F | CGGCCCCGACTGCGGTTCTGTCT | Col2a1-F | AGGATGTATGGAAGCCCTCATCTTGC |
| Ihh-R | ACGAGCTTGCGAGGCGGCCT | Col2a1-R | GGGTTGAGGCAGTCTGGGTCTT |
| BDNF-F | GACTCTGGAGAGCGTGAATGGG | Mmp13-F | TCATACTACCATCCTGCGACTCTTGC |
| BDNF-R | GCAGCTCTTCGATGACGTGC | Mmp13-R | GCCAGTCACCTCTAAGCCAAAGAAAG |
