# Supplementary figures and images for: Trp53 controls chondrogenesis and endochondral ossification by negative regulation of TAZ activity and stability via β-TrCP-mediated ubiquitination
Source: Cell Death Discov. 2022 Jul 12;8:317. doi: 10.1038/s41420-022-01105-2 (PMC9279315; doi:10.1038/s41420-022-01105-2)

Original western blots

Fig. 1E

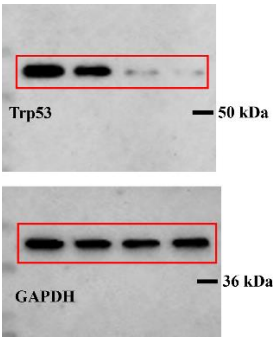

Fig. 5C

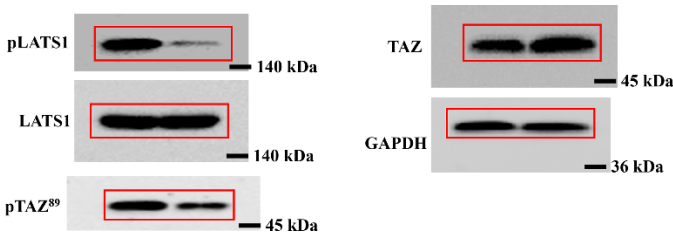

Fig. 5D

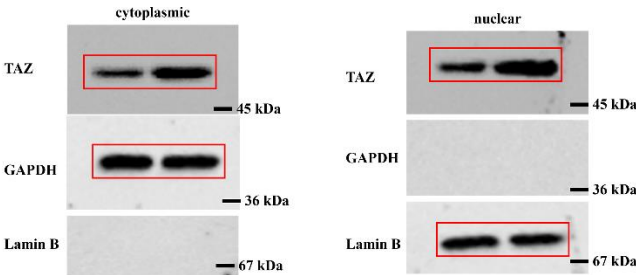

**Fig. 5H**

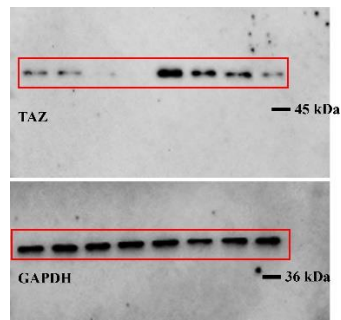

**Fig. 5I**

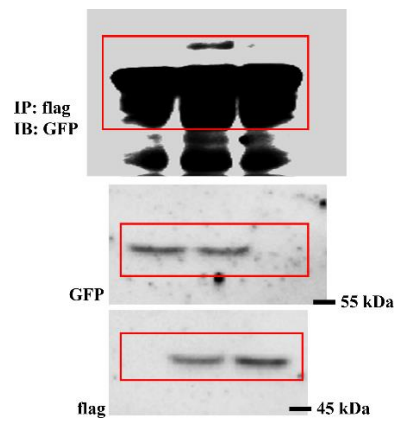

**Fig. 5J**

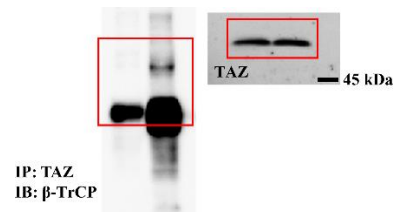

**Fig. 5K**

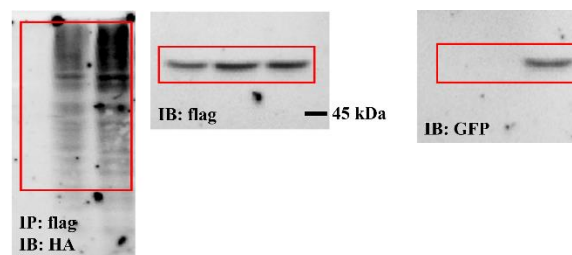

**Fig. S1C**

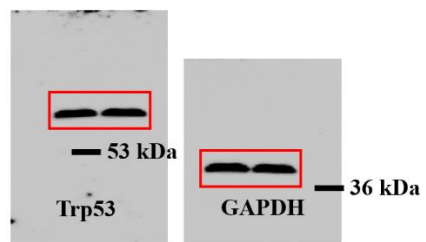

Supplement: Supplementary file 2 — Original Data File [file 41420_2022_1105_MOESM2_ESM.pdf]

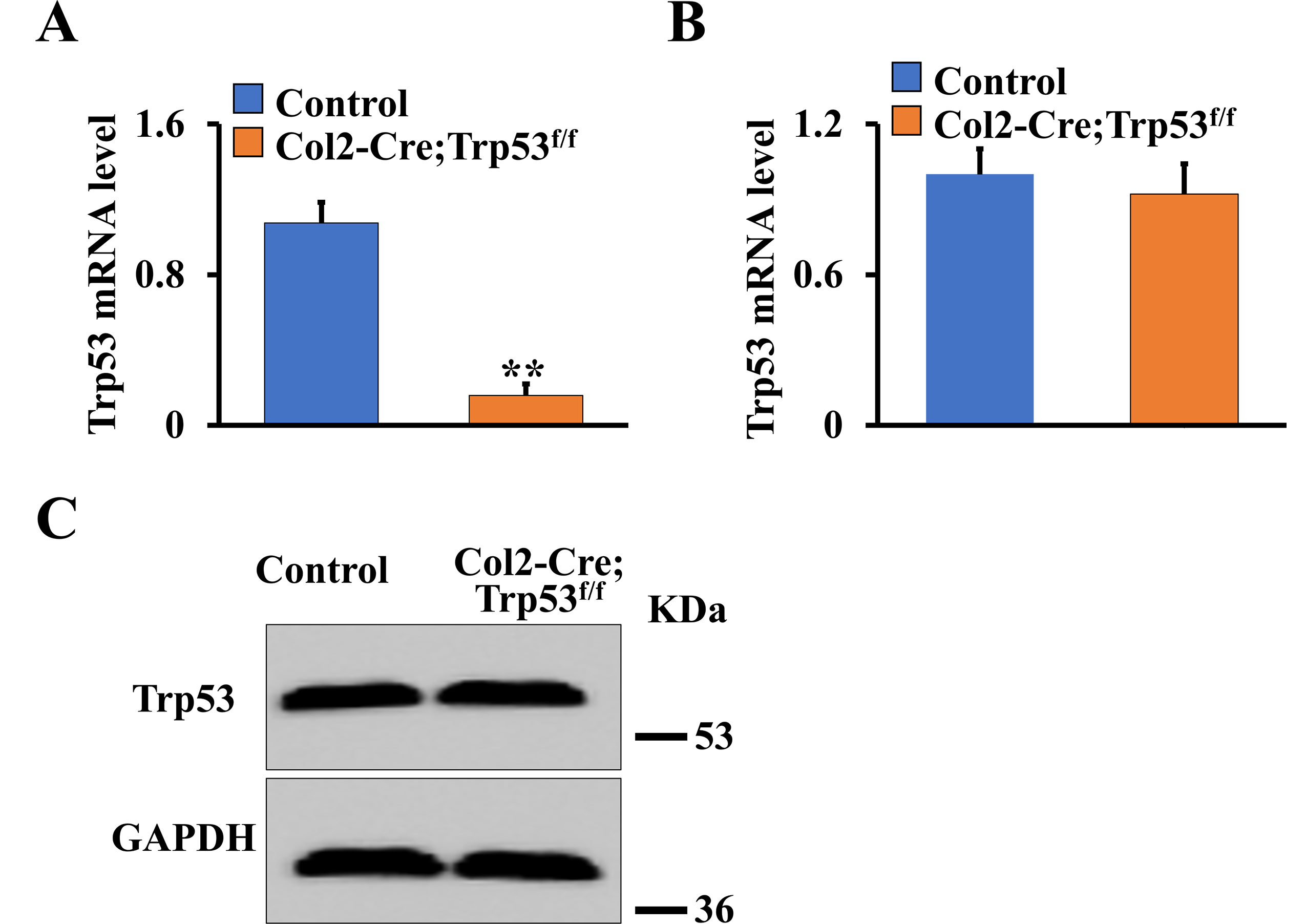

Supplement: Supplementary file 3 — Figure S1 [file 41420_2022_1105_MOESM3_ESM.tif]

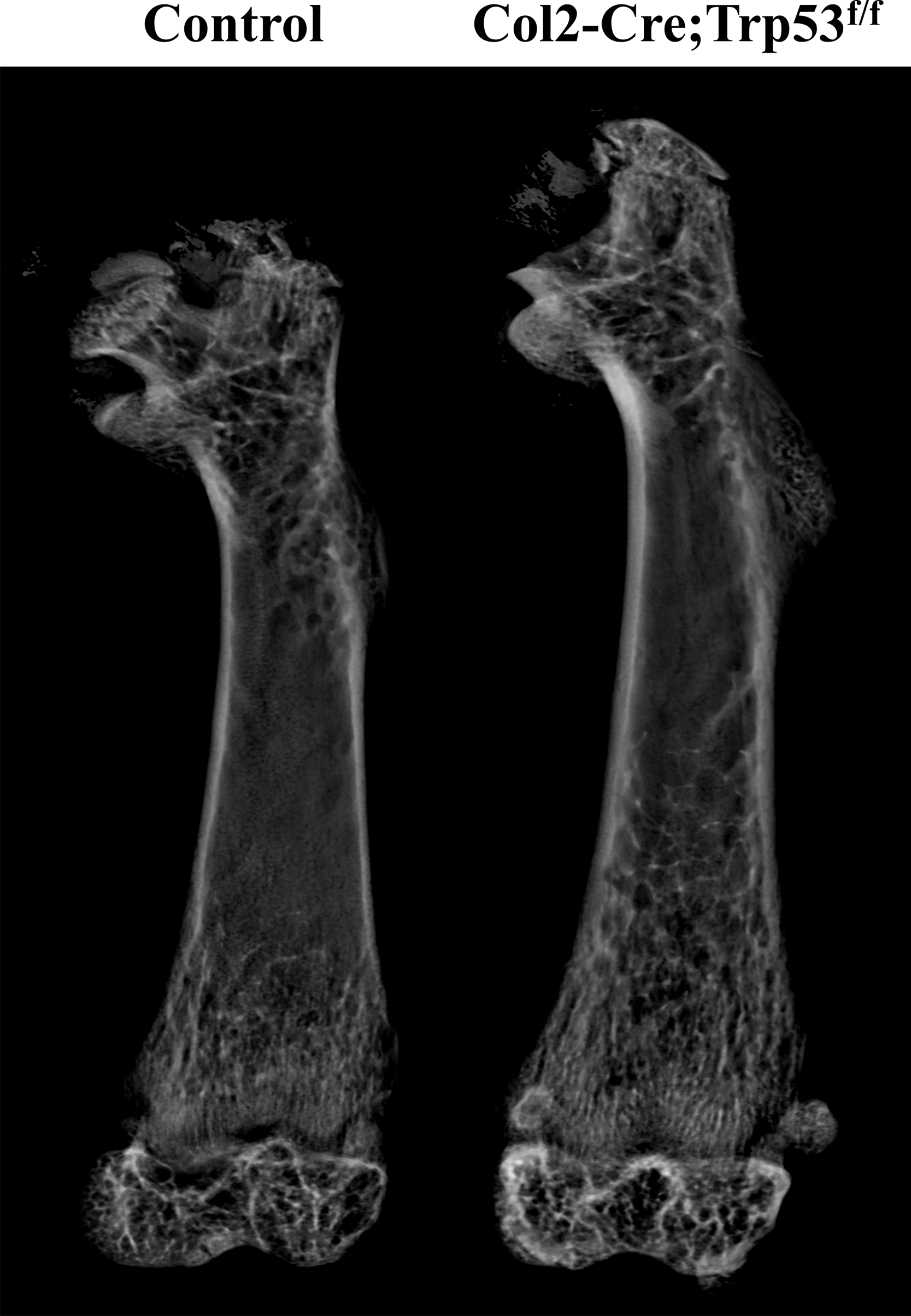

Supplement: Supplementary file 4 — Figure S2 [file 41420_2022_1105_MOESM4_ESM.tif]
